# SRM-MS Method Development for Hepcidin-25 Peptide

**DOI:** 10.1155/2018/9653747

**Published:** 2018-06-14

**Authors:** Kyung-Cho Cho, Byoung-Kyu Cho, Jin Woo Jung, Ye Ji Lee, Eun Bong Lee, Eugene C. Yi

**Affiliations:** ^1^Department of Pathology, Johns Hopkins University, Baltimore, MD 21287, USA; ^2^Department of Molecular Medicine and Biopharmaceutical Science, Graduate School of Convergence and Technology, School of Medicine of School of Pharmacy, Seoul National University, Seoul, Republic of Korea; ^3^Division of Rheumatology of Internal Medicine, Seoul National University College of Medicine, Seoul, Republic of Korea; ^4^Graduate Course of Translational Medicine (Immunology), Seoul National University College of Medicine, Seoul, Republic of Korea; ^5^Department of Biomedical Engineering, Seoul National University Hospital, Seoul, Republic of Korea

## Abstract

As advanced mass spectrometry- (MS-) based hepcidin analysis offers to overcome the limitations in analytical methods using antihepcidin, further improvement of MS detection sensitivity for the peptide may enhance the diagnostic value of the hepcidin for various iron-related disorders. Here, improved MS detection sensitivity of hepcidin has been achieved by reducing the disulfide bonds in hepcidin, by which proton accessibility increased, compared to the intact hepcidin peptide. Comparing the ionization efficiencies of reduced and nonreduced forms of hepcidin, the reduced form of hepcidin showed an increase in ionization efficiency more than two times compared to the nonreduced form of hepcidin. We also demonstrated improved detection sensitivity of the peptide in SRM assay. We observed a significant improvement of detection sensitivity at the triple-quadrupole MS platform, that the ionization efficiency increased at least twice more, and that the limit of detection (LOD) increased more than 10 times in the concentration ranges of 1 fmol to 10 fmol of hepcidin. In this study, we demonstrated the usefulness of the hepcidin modification for overall enhancement of the ionization efficiencies of the hepcidin peptide in the MS-based quantitative measurement assay.

## 1. Introduction

Hepcidin, a folded 25-residue peptide hormone stabilized by four disulfide bonds, plays an important role in the regulation of iron metabolism in mammals, such as digestive iron absorption and macrophage iron recycling [1–3]. Although anemia of inflammation and chronic kidney diseases are developed in various clinical settings, the most direct causes of the diseases are associated with the destruction of homeostasis of iron metabolism [4–6]. Hence, hepcidin has been considered as an important clinical utility for the diagnosis and management of a wide range of iron-related disorders [7–10]. Quantitative immunoassays based on the use of antihepcidin antibodies have been developed for quantitative assessment of hepcidin concentrations in the patient's serum and urine [11–15]. However, the antibody-based immunoassays have the limitations because it is interfered by *N*-terminal deletion forms as well as isoforms such as prohepcidin and preprohepcidin [16, 17]. Recent advances in mass spectrometry- (MS-) based quantitative assays (e.g., SRM assay) for hepcidin complement the immunoassays by overcoming hepcidin (25-amino acids) detection specificity, reproducibility, and accuracy [18–22]. In addition, absolute concentrations of hepcidin can be measured with synthetic hepcidin labeled with heavy stable isotopes in SRM assay [23, 24].

Further improvement of MS detection sensitivity for hepcidin would increase its clinical utility. Peptide MS ionization capability is mainly dependent on its amino acid composition, such as hydrophilicity or hydrophobicity, presence or absence of a chemical modification, and protonation capability. Intradisulfide bonds of hepcidin interrupt the proton accessibility to the peptide, which decreases the peptide ionization efficiency in MS analysis [25, 26]. Reduction of disulfide bonds unfolds the protein/peptide structure and allows more proton accessibility to the peptides for protonation, which increases the peptide charge states.

In this study, we improved the ionization efficiency of intact hepcidin by reducing intradisulfide bonds in hepcidin. To validate the sensitivity enhancement of reduced form of hepcidin, we performed SRM assay with optimized peptide transitions and determined its LOD, thus validating the method for measurement of hepcidin.

## 2. Materials and Methods

### 2.1. Materials

Human hepcidin-25 peptide (DTHFPICIFCCGCCHRSKCGMCCKT, SML1118, Sigma-Aldrich, purify >95%, 4 disulfide bonds; C7-C23, C10-C13, C11-19, and C14-C22), stable isotope- (SI-) labeled human hepcidin-25 peptide, ([^13^C_9_/^15^N]Phe4,9,[^15^N]Gly12, PLP-3405-v, Peptide Institute Inc., Osaka, Japan), dithiothreitol (DTT), iodoacetamide (IAA), and ammonium bicarbonate were purchased from Sigma-Aldrich (St. Louis, MO, USA). C18 spin column (Cat no. #89870) was purchased from Pierce (Rockford, IL, USA). HPLC-grade water and acetonitrile were purchased from Burdick & Jackson (Philipsburg, NJ, USA).

### 2.2. Reduction and Alkylation of Hepcidin

For reducing the intradisulfide bonds in hepcidin peptide, the peptide was incubated with 10 mM DTT in 50 mM ammonium bicarbonate (pH 7.5) for 30 min at 37°C, and cysteine alkylation was performed with 55 mM IAA for 30 min at RT in dark. Peptides were purified using a C18 spin column prior to LC-MS/MS analysis.

### 2.3. Extraction of Serum Hepcidin Peptides

Two 100 *µ*L of human serum samples containing 50 pmol of SI-hepcidin were prepared. Hepcidin peptides were extracted with 90% ACN in 0.1% formic acid in water after incubation for 20 min at 4°C. After drying the supernatants, one of the extracted hepcidin peptide samples was reduced and alkylated with DTT and IAA. Hepcidin peptides were desalted prior to the SRM assay, and only 1/10 of desalted sample was used for analysis. The serum hepcidin was extracted from the serial serum samples (100, 200, 400, and 800 *μ*L) with 100 pmol of SI-hepcidin spiked-in.

### 2.4. LC-MS/MS Analysis

Both intact and modified hepcidin peptides were analyzed using Q Exactive™ mass spectrometer (Thermo Fisher Scientific Inc., Germany) interfaced with easy-nLC1000 (Thermo Fisher Scientific, Waltham, MA, USA) as described previously [27]. Equal amount hepcidin peptides were loaded onto a precolumn (PepMap, 75 *μ*m ID  *μ*m*∗*2 cm 3 *μ*m, 164535, Thermo Fisher Scientific, USA) and separated using an analytic column (PepMap, 75 *μ*m ID*∗*50 cm 3 *μ*m, ES803, Thermo Fisher Scientific, USA). The sample was eluted by solvent A (0.1% formic acid in water) and solvent B (0.1% formic acid in acetonitrile). The mobile phase gradient parameter was set follow as: time (B%) 0∼3 min (5% solvent B), 50 (40%), 55 (80%), 57 (80%), 60 (5%), and 70 (5%) at a flow rate of 300 nL/min. LC-MS/MS data were acquired on a mass resolution of 70 K at 200 m/z using Q Exactive mass spectrometer. The instrument was operated in data-dependent mode; top 10 intensity precursor ions were selected for subsequent MS/MS analysis by HCD with a normalized collision energy (NCE) value of 27 and, resolution of MS/MS was set as 17 K at 200 m/z.

### 2.5. SRM Analysis

SRM analysis was performed using an Agilent 6490 triple quadrupole mass spectrometer (Agilent Technologies, USA) interfaced with an Agilent 1290 Infinity System (Agilent Technologies, USA). Peptides were eluted by reverse-phase UPLC on a Zorbax Eclispse Plus C18 column (150 × 2.1 mm, 1.8 um particle size, Agilent Technologies, USA) at 0.25 mL/min over a 30 min ACN gradient. The column temperature was maintained at 50°C. The gradient was set as follows using mobile phases A (0.1% formic acid in water) and B (0.1% formic acid and 5% water in ACN): linear 0–40% B for 20 min, 40∼90% B for 20.1 min, isocratic 90% B for 5 min, linear 90–0% B for 0.1 min, and isocratic 0% B for 5 min. The AJS ESI voltage was set as 3500 V with a gas flow of 12 L/min, source temperature of 250°C, and fragmentor voltage of 5. Each scan was collected by dwell time of 20 ms.

## 3. Results

### 3.1. Characterization of the Modified Hepcidin

**Figure 1:**
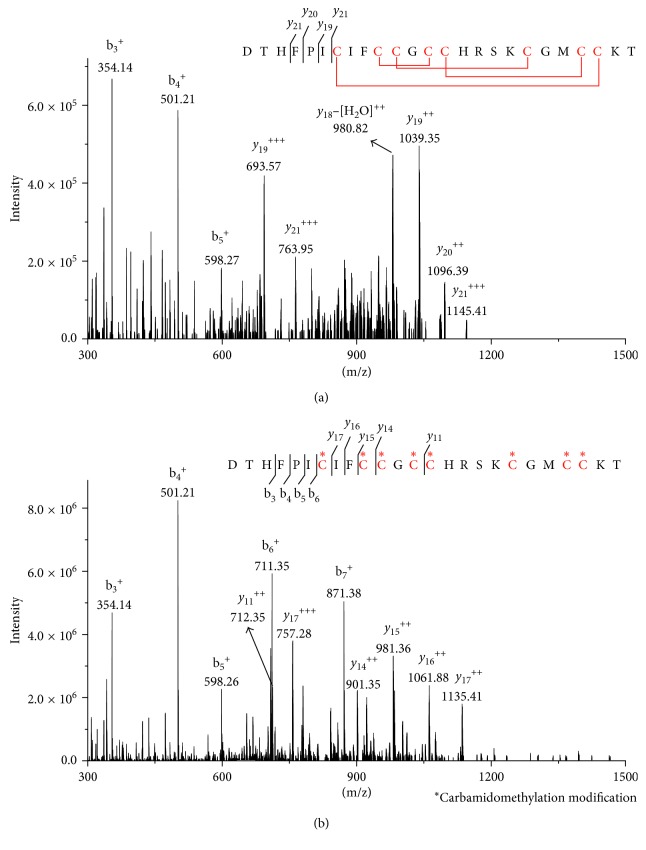
MS/MS spectrum of hepcidin-I and hepcidin-M. (a) is the MS2 spectrum for 558.6 m/z (*z* = 5) derived from the hepcidin-I, and (b) is for 651.8 m/z (*z* = 5) derived from the hepcidin-M. The cysteine (C) amino acid sequence of red color indicates disulfide bond and carbamidomethylation position in (a) and (b).

To improve MS detection sensitivity of hepcidin, the disulfide bonds of intact hepcidin peptide (hepcidin-I) was reduced, and the resulting free thiols were subsequently modified by alkylation to linearize the peptide (hepcidin-M) as described in Materials and Methods. To verify the thiol modification of hepcidin-I, a mixture of equal amounts (10 pmol) of both peptides were analyzed by LC-MS/MS. As shown in Figure 1, a series of *y* ions that are corresponding to peptide fragments of nonreduced cysteine residues are observed (Figure 1(a)), whereas the MS/MS spectrum of hepcidin-M shows a series of *b* and *y* ions that are matched to the reduced forms of cysteine residues, indicating that all the disulfide bonds were reduced and alkylated (Figure 1(b)).

**Figure 2:**
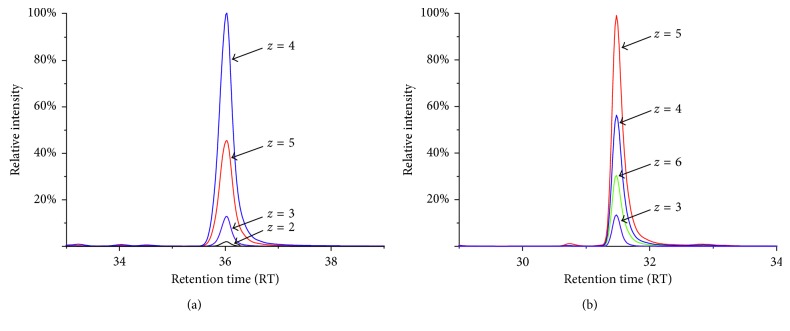
Extracted ion chromatograms of hepcidin-I and hepcidin-M according to charge state. (a) A hepcidin-I. (b) A hepcidin-M.

MS ionization efficiencies of both peptides were verified by comparing their signal intensities at different charge states of Hepcidin-I (m/z, 2787.0258 Da) and hepcidin-M (m/z, 3251.2602 Da). This experiment was conducted to select the most intense signal intensities of peptide charge states for the purpose of the hepcidin LC/MS assay development.  Figure 2 shows extracted ion chromatograms (XIC) for relative abundances of different charge states of Hepcidin-I (*z* = 2, 3, 4, and 5) (Figure 2(a)) and of Hepcidin-M (*z* = 3, 4, 5, and 6) (Figure 2(b)). Among these charge states, it appears that the +4 charge state of Hepcidin-I shows the highest peptide signal intensity, whereas hepcidin-M shows the most intense peptide signal when it is in the +5 charge state. Hepcidin-M tends to give more intense signal intensities with higher charge states, indicating that its linearization may increase the proton accessibility. The reduced form of hepcidin decreases the peptide hydrophobicity, which exhibits its reversed-phase HPLC elution time of about 4 minutes faster than hepcidin-I in the given HPLC condition.

### 3.2. Ionization Efficiency of Hepcidin-M

Typically, ionization efficiency of peptides under ESI condition is affected by the proton accessibility and hydrophobicity of peptide analytes [28, 29]. As shown in Figure 2, cysteine reduction and alkylation of hepcidin peptide modify the property such as reduction of hydrophobicity and leading to increased proton accessibility of native form of hepcidin peptide. To quantitatively compare ionization efficiencies of both hepcidin related to their charge states and relative ionization (RI) of the peptide at each charge state is calculated by the following equation: RI (*n*) = XIC area of Hepcidin -M^*n*+^/XIC area of Hepcidin -I^*n*+^ (*n*=2 ~ 6), where n is a charge state, and XIC area is obtained from ESI mass spectrometry analysis (Q Exactive).

As shown in Table 1, the relative ionization efficiency of hepcidin-M is higher than hepcidin-I (2-fold to as high as 40-fold) in most of their charge states, except the +4 charge states, indicating that the relative ionization efficiency of hepcidin enhanced by the derivatization.

### 3.3. SRM Assay Optimization

To further demonstrate quantitative assessment of hepcidin by a targeted MS approach, peptide transitions of hepcidin-I and hepcidin-M were optimized for SRM assay using triple quadrupole mass spectrometer (Agilent 6490 QQQ). “Full scan mode” was performed to identify peptide precursor (MS^1^) ions for both hepcidin peptides. Similar charge state-dependent peptide ionization was observed between the two MS platforms (QQQ and Q Exactive). Both hepcidin-I and hepcidin-M produced intense MS1 peptide signals when they were at +4 and +5, respectively (data not shown). To select the optimal fragment (MS^2^) ions for both peptides, “product ion scan” was performed by varying m/z having different charge states and collision energy (Supplementary Materials Figure 1). Although the hepcidin-I showed the highest signal intensity of MS^1^ ions at +4 charge state, the +5 charge state of MS1 of the peptide generated more intense MS^2^ ion intensities (5.3 × 10*E*4 (*y*_19_^+++^, at collision energy 15 V) and 1.3 × 10*E*4 (*y*_21_^+++^, at 15 V)) than the +4 charge state peptide (1.2 × 10*E*4 (*y*_19_^+++^, at 25 V) and 8 × 10*E*3 (*y*_21_^+++^, 25 V)). The two highest *y* ions of hepcidin-M were 2.6 × 10*E*5 (*y*_17_^+++^, at 15 V) and 1.6 × 10*E*5 (*y*_16_^+++^, at 15 V) at the +5 charge state. These MS^2^ ions were selected for the SRM assay optimization. Further collision energy optimization was automatically selected using “Peptide Optimizer” software. The optimized peptide transitions are summarized in Table 2.

**Figure 3:**
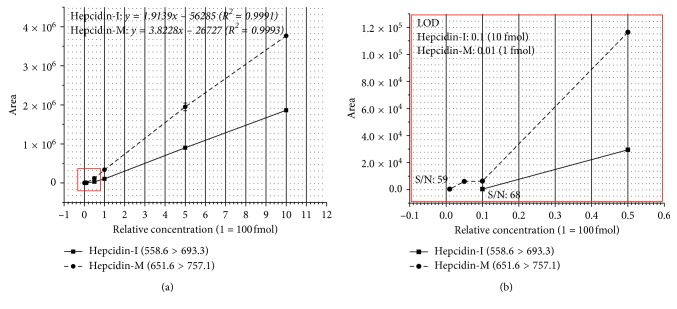
The graph shows the peak area for hepcidin amount ranging from 1 fmol to 1 pmol (1, 5, 10, 50, 100, and 500 fmol and 1 pmol). The graph of hepcidin-I and -M were presented using 558.6 > 693.3 and 651.6 > 757.1 of transitions, respectively. (a) R2 of hepcidin-I and -M were obtained by the equations of *y* = 1.9139*x*−56285 and *y* = 3.8228*x*−26727, respectively. (b) An enlarged graph of the red box portion in (a), showing the peak area according to the amount of 1 fmol to 50 fmol and S/N value of LOD for both hepcidin-I and hepcidin-M.

To evaluate the detection sensitivity in SRM assay for both hepcidin-I and hepcidin-M with optimized peptide transition ions, three replicate analysis for both peptides were conducted in the concentration ranges from 1 fmol to 1 pmol (1, 5, 10, 50, 100, and 500 fmol and 1 pmol) using Agilent 6490 QQQ. Peptide transitions (peak area) were calculated using Skyline (MaCcoss lab software) [30]. Among all the peptide transitions, the highest peptide transition values were selected and the average value of three replications was calculated. Quantitative linearity curves (concentration *vs* calculated peak area ratios) for both peptides are quantitatively linear. As shown in Figure 3(a), *R* ^2^ values were obtained from each linear curve and estimated for hepcidin-I and -M as 0.9991 and 0.9993, respectively. At concentration of 50 and 100 fmol, peak area of hepcidin-M (1.95 × 10^6^ and 3.77 × 10^6^) is at least twice more than that of hepcidin-I (9.00 × 10^5^ and 1.86 × 10^6^). At lower concentration, the peak area of 10, 50, and 100 fmol of hepcidin-M were 6.31 × 10^3^, 1.16 × 10^5^, and 3.36 × 10^5^ and of hepcidin-I were 3.90 × 10^2^, 2.94 × 10^4^, and 1.05 × 10^5^, suggesting that the detection sensitivity of hepcidin-M is better than hepcidin-I. The limit of detection (LOD) of each peptide was determined by S/N (signal-to-noise ratio) value of 10 or more. As shown in Figure 3(b), the LODs of hepcidin-I and -M were 10 and 1 fmol, respectively. Hepcidin-I showed the S/N value of 10 more (S/N: 69) at 100 fmol but showed 10 less at 10 and 50 fmol (data not shown), while hepcidin-M showed the S/N value of greater than 10 at 1 fmol (S/N: 58). These results also indicate that the LOD of hepcidin peptide improved 10-fold after reduction and alkylation modification in SRM assay optimization.

**Figure 4:**
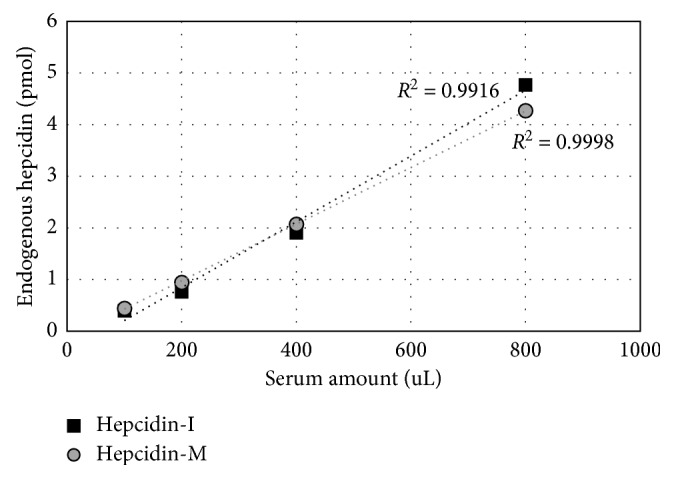
The graph shows correlation between serum amount (*μ*L) and endogenous hepcidin (pmol). Hepcidin amount was calculated using the peak area of SI-hepcidin (100 pmol) acquired from SRM analysis.

The improved hepcidin peptide detection sensitivity by the reduction/alkylation modification was further verified with human serum samples. Extracted serum hepcidin peptides spiked with equal amounts of SI-hepcidin were quantitatively measured by the SRM analysis. The results of the SRM analysis showed that the relative peak ratio between the endogenous hepcidin-M and SI-hepcidin-M was 0.01 (50 fmol of serum hepcidin in the 10 *μ*L of serum sample), whereas the relative peak ratio for hepcidin-I could not be determined, indicating that serum hepcidin was detected only with the reduced hepcidin form (Table 3). Endogenous hepcidin-I and hepcidin-M levels were quantitatively measured in the serial serum samples (100, 200, 400, and 800 *μ*L) with 100 pmol of SI-hepcidin spiked-in. Equal of amount of SI-hepcidin (100 pmol) were spiked into the serum samples (100 to 800 *μ*L). Endogenous hepcidin-I and -M levels were calculated based on the ratio between endogenous- and SI-hepcidin showing the quantitative correlation (Figure 4 and Supplementary Materials Figure 2).

## 4. Discussion

Hepcidin has shown to have an important clinical utility for the diagnosis and management of a wide range of iron-related disorders, the diagnostic value for various iron-related disorders. Although many analytical methods including antibody-based assays have been developed to measure the hepcidin p level, quantitative assessment of hepcidin using SRM-MS has emerged as a promising quantitative technique to measure the hepcidin level owing to the numerous advantages of mass spectrometry-based analytical method. In order to improve the hepcidin detection sensitivity SRM assay, we derivatized the intradisulfide bonds in hepcidin using the DTT and IAA reagents, which are the most common chemical reactions to reduce the disulfide bonds. In this experiment, modification of the hepcidin showed a substantial increase in ionization efficiency of more than 2 times. The LOD value is 10 times lower than that of the conventional one by the modification. Spiking of human serum samples with a known amount of stable isotope-labeled synthetic hepcidin peptide allows measurement of the corresponding hepcidin peptide present in the serum sample.

In blood and urine, hepcidin is present as the form of hepcidin-25 as well as in truncated form of hepcidin isoforms, hepcidin-20, and hepcidin-22 [31]. These truncated hepcidin isoforms are mainly in the blood of patients with sepsis-induced acute kidney failure; however, it is difficult to detect because the amount of those hepcidin isoforms is much less than that of hepcidin-25. Although biological samples such as blood and urine are noninvasive sample sources, collecting large quantities is still a burden on patients. Hence, we anticipate that hepcidin measurement using the improved SRM assay may require an ample amount of samples from patients than the current methods of hepcidin measurement.

## 5. Conclusion

In this study, we demonstrate the developmental SRM assay for hepcidin. By simply reducing disulfide bond in hepcidin, ionization efficiency has been increased at least 2 folds more. Modified hepcidin also has been shown that sensitivity in SRM assay increased 10 folds more than unmodified form. We also further demonstrated the improvement of serum hepcidin peptide detection sensitivity by the reduction/alkylation modification and quantitative similarity with hepcidin-I in the SRM assay. This study provides improved detection sensitivity for hepcidin, which may increase its clinical utility for the diagnosis and management of a wide range of iron-related disorders.

## Figures and Tables

**Table 1:** Relative ionization value of hepcidin-M to hepcidin-I according to charge state.

Charge state	Hepcidin-I		Hepcidin-M		Relative ionization value (hepcidin-M/I)
	m/z	Area	m/z	Area	
2	1394.52	9.7*E* + 07	1626.64	3.8*E* + 08	3.92
3	930.02	1.3*E* + 09	1084.76	3.0*E* + 09	2.34
4	697.76	9.0*E* + 09	813.82	8.4*E* + 09	0.93
5	558.41	8.7*E* + 09	651.26	2.3*E* + 10	2.69
6	465.51	8.9*E* + 07	542.88	3.7*E* + 09	41.76
Sum of area		1.9*E* + 10		3.9*E* + 10	2.03

**Table 2:** A transition list for SRM assay of each of the hepcidin-I and -M containing precursor ion, product ion, and CE values.

Peptide sequence	Precursor ion	Product ion	Dwell time	Collision energy	Fragment
DTHFPICIFCCGCCHRSKCGMCCKT	558.6	763.3	20	16	y21^3+^
DTHFPICIFCCGCCHRSKCGMCCKT	558.6	693.3	20	14	y19^3+^
DTHFPIC*∗*IFC*∗*C*∗*GC*∗*C*∗*HRSKC*∗*GMC*∗*C*∗*KT	651.6	757.1	20	14	y17^3+^
DTHFPIC*∗*IFC*∗*C*∗*GC*∗*C*∗*HRSKC*∗*GMC*∗*C*∗*KT	651.6	708.4	20	14	y16^3+^

**Table 3:** Relative ratio of peak area between serum hepcidin-M and SI-hepcidin-I.

	Peak area		Relative ratio (endogenous/isotope hepcidin)
	Endogenous	Stable isotope	
Hepcidin-I	N/A	12105	N/A
Hepcidin-M	301	30542	0.01

## Supplementary Materials

Supplementary 1 Figure 1: MS/MS spectra according to different collision energy (CE) values. The red lettering in the spectral figure represented the *y* fragment ion selected for MRM assay. (a) and (b) are the MS/MS spectra of the 698.0 and 558.6 m/z derived from hepcidin-I. (c) and (d) are MS/MS spectra of 543.0 and 651.8 m/z derived from hepcidin-M.

Supplementary 2 Figure 2: the graphs show the extracted ion chromatography (EIC) of hepcidin-I (a) and hepcidin-M (b) obtained from SRM assay using 800 *μ*L of serum sample. Each EIC shows the peak intensity of endogenous- and SI-hepcidin (left) and also shows the peak intensity according to transitions (right).
