# Supplementary material for: SRM-MS Method Development for Hepcidin-25 Peptide
Source: J Anal Methods Chem. 2018 Jun 14;2018:9653747. doi: 10.1155/2018/9653747 (PMC6022310; doi:10.1155/2018/9653747)

Figure legends

Supporting Information Figure 1. MS/MS spectra according to different collision energy (CE) values. The red lettering in the spectral figure represented the y fragment ion selected for MRM assay. (A) and (B) MS/MS spectra of the 698.0 and 558.6 m/z derived from hepcidin-I. (C) and (D) MS/MS spectra of 543.0 and 651.8 m/z derived from hepcidin-M.

Supporting Information Figure 1

(A) Hepcidin-I “Product ion scan” 698.0 m/z ($[M+4]^{4+}$) >

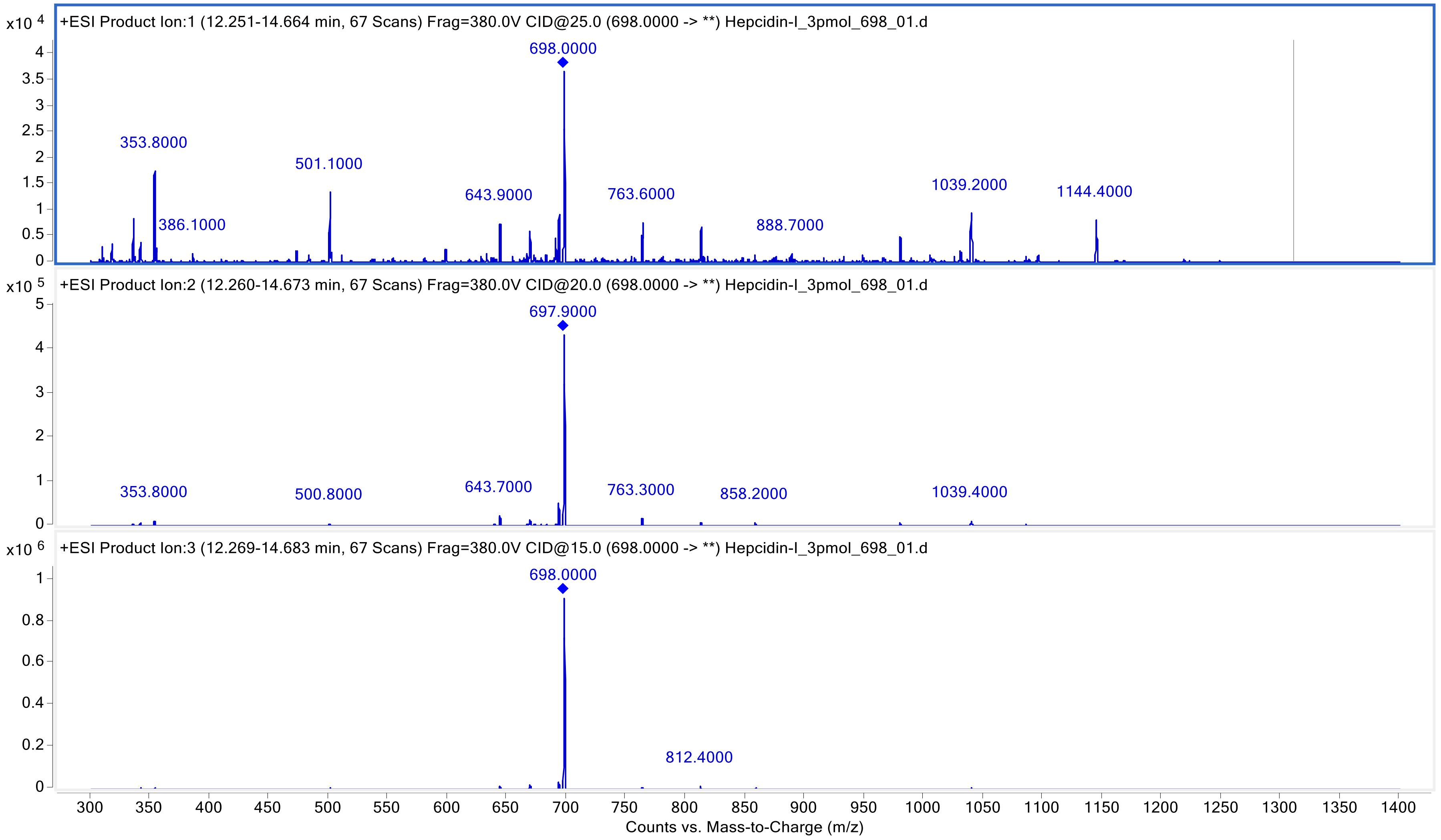

Supporting Information Figure 1

(B) Hepcidin-I "Product ion scan" 558.6 m/z ([M+5]$^{5+}$) >

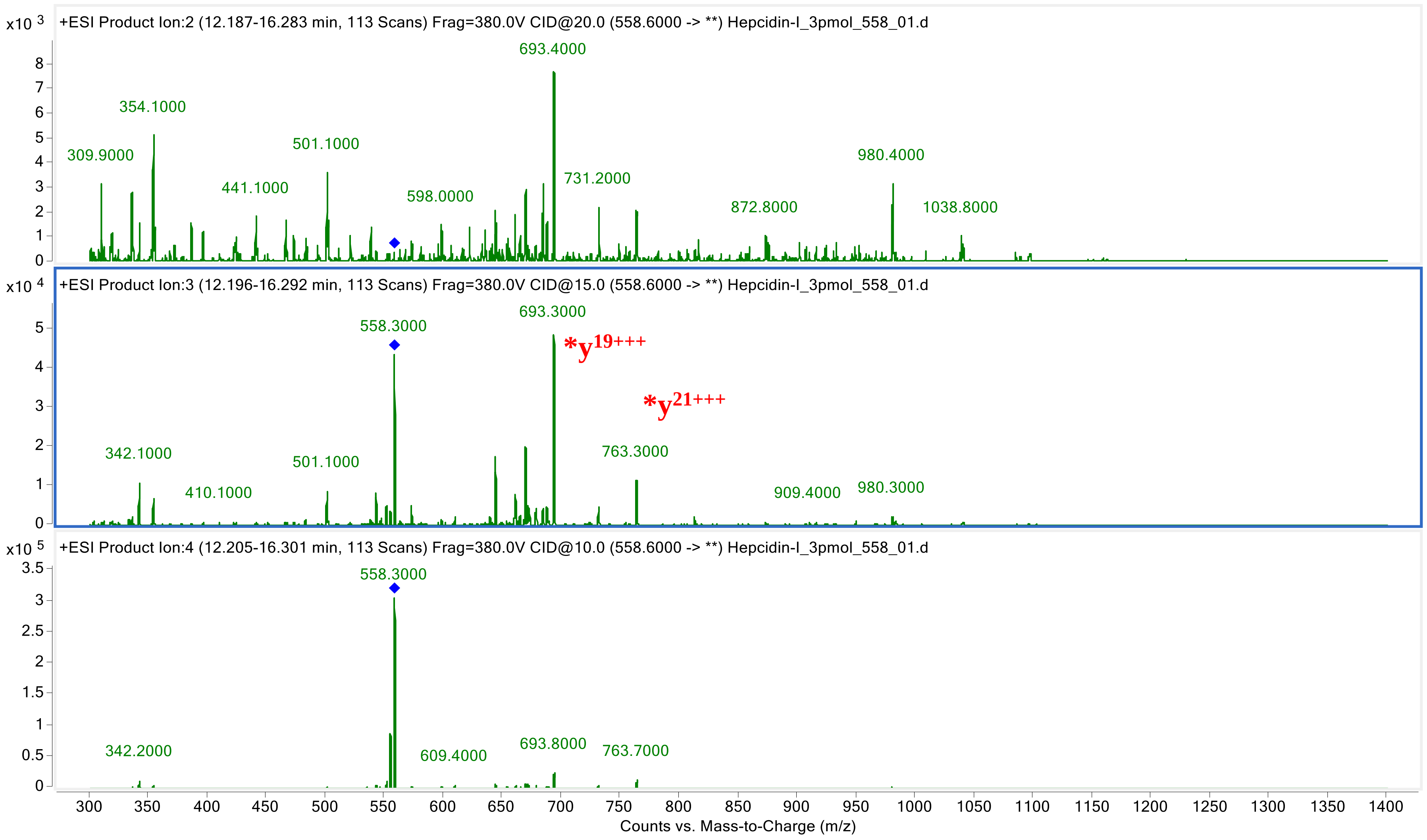

Supporting Information Figure 1

(C) Hepcidin-M “Product ion scan” 651.8 m/z ($[M+5]^{5+}$) >

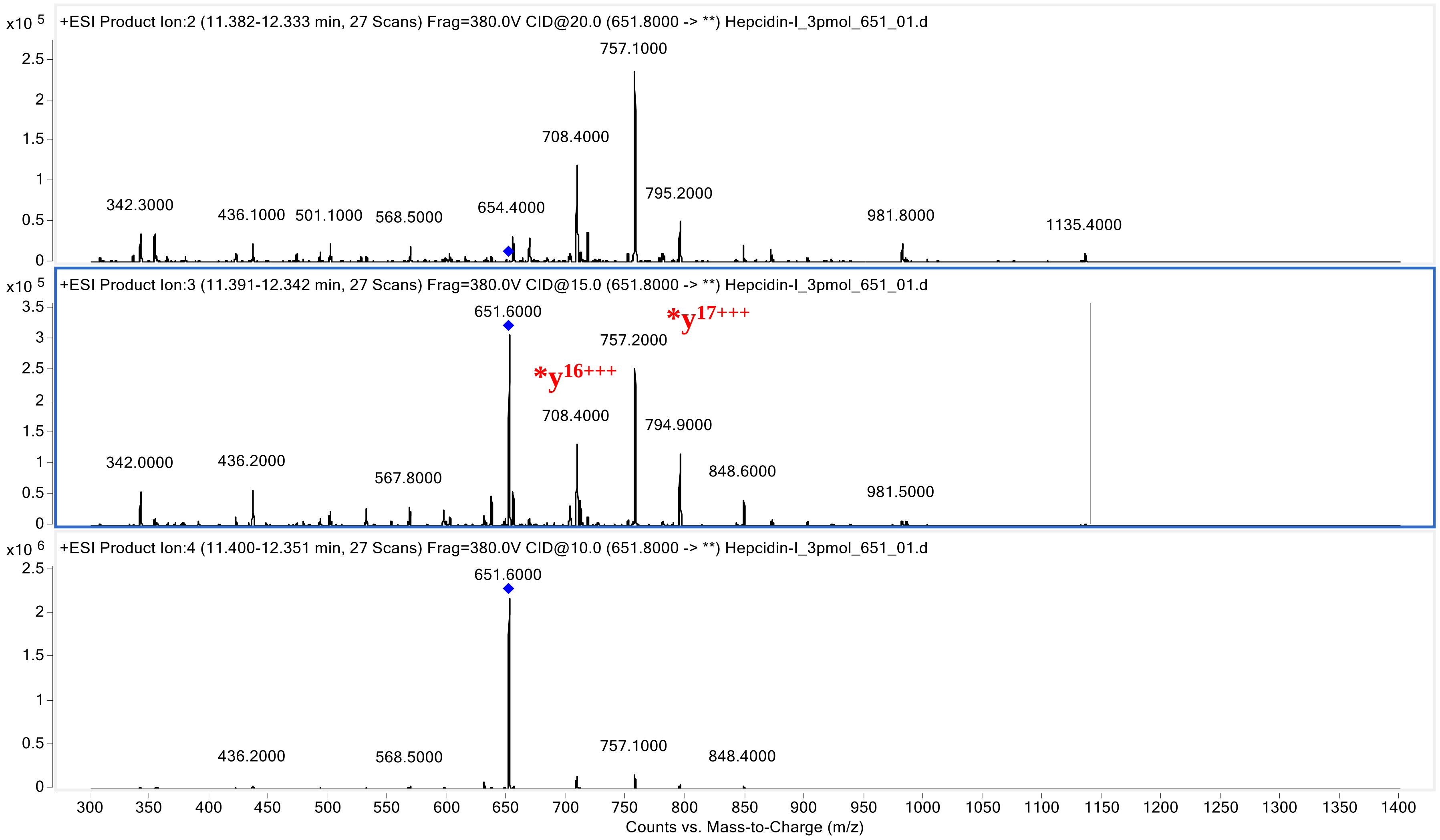

Supporting Information Figure 1

(D) Hepcidin-M "Product ion scan" 543.0 m/z ([M+6]$^{6+}$) >

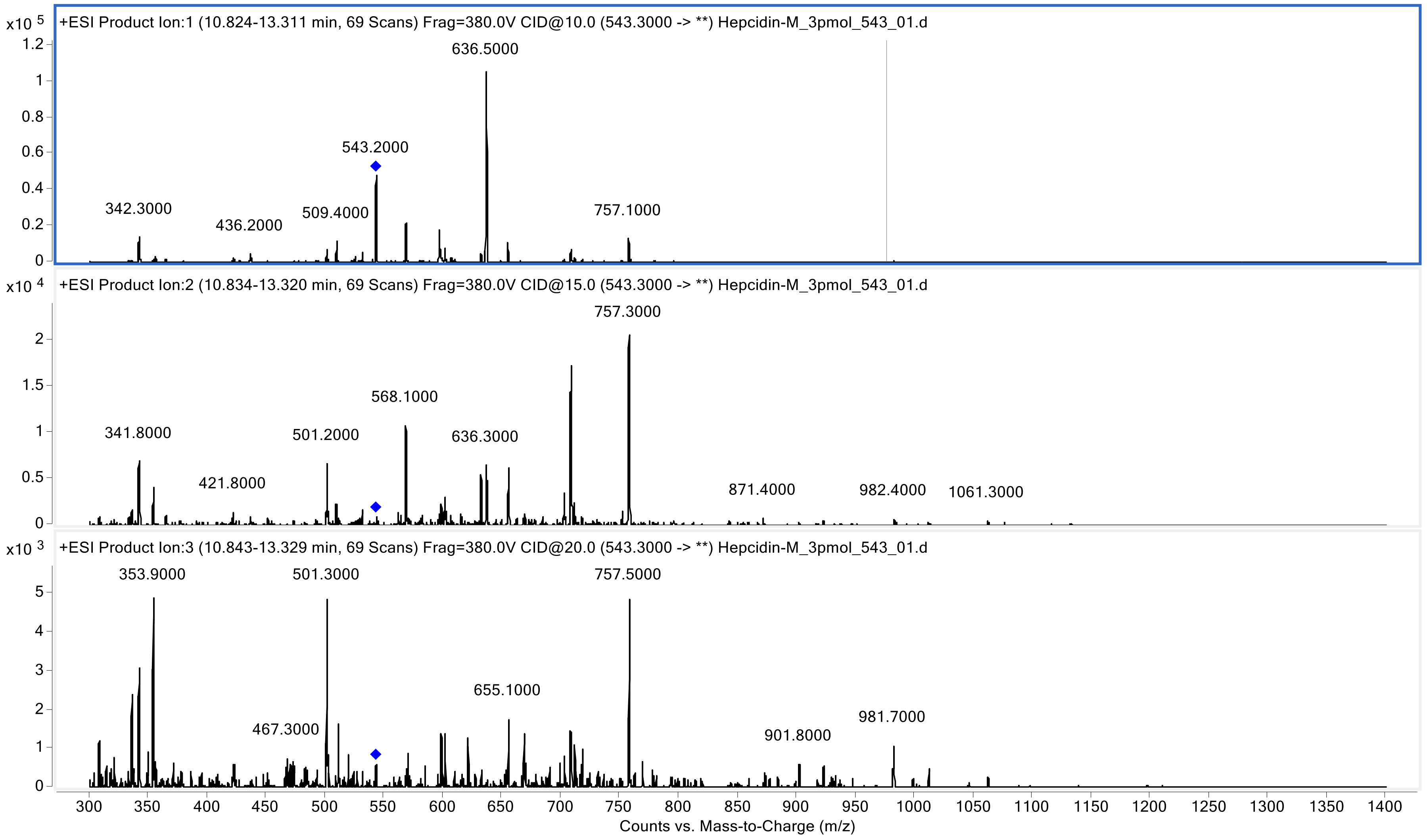

Supplement: Supplementary 1 — Figure 1: MS/MS spectra according to different collision energy (CE) values. The red lettering in the spectral figure represented the y fragment ion selected for MRM assay. (a) and (b) are the MS/MS spectra of the 698.0 and 558.6 m/z derived from hepcidin-I. (c) and (d) are MS/MS spectra of 543.0 and 651.8 m/z derived from hepcidin-M. [file 9653747.f1.pdf]
