# Supplementary material for: SRM-MS Method Development for Hepcidin-25 Peptide
Source: J Anal Methods Chem. 2018 Jun 14;2018:9653747. doi: 10.1155/2018/9653747 (PMC6022310; doi:10.1155/2018/9653747)

Figure legends

Supporting Information Figure 2. The graphs show the extracted ion chromatography (EIC) of hepcidin-I (A) and hepcidin-M (B) obtained from SRM assay using 800 μL of serum sample. Each EIC shows the peak intensity of endogenous- and SI-hepcidin (left) and also shows the peak intensity according to transitions (right).

Supporting Information Figure 2

(A)

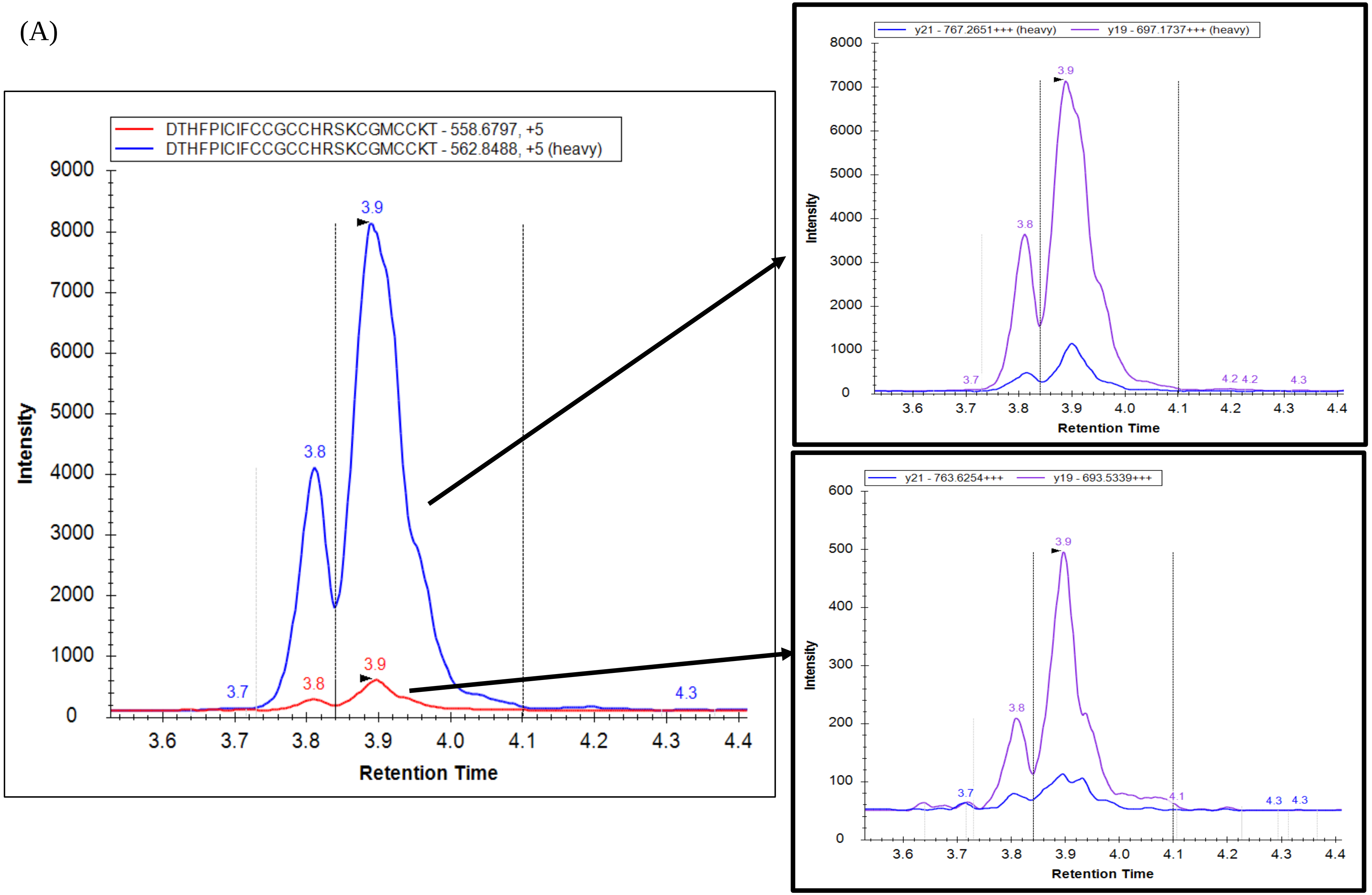

Supporting Information Figure 2

(B)

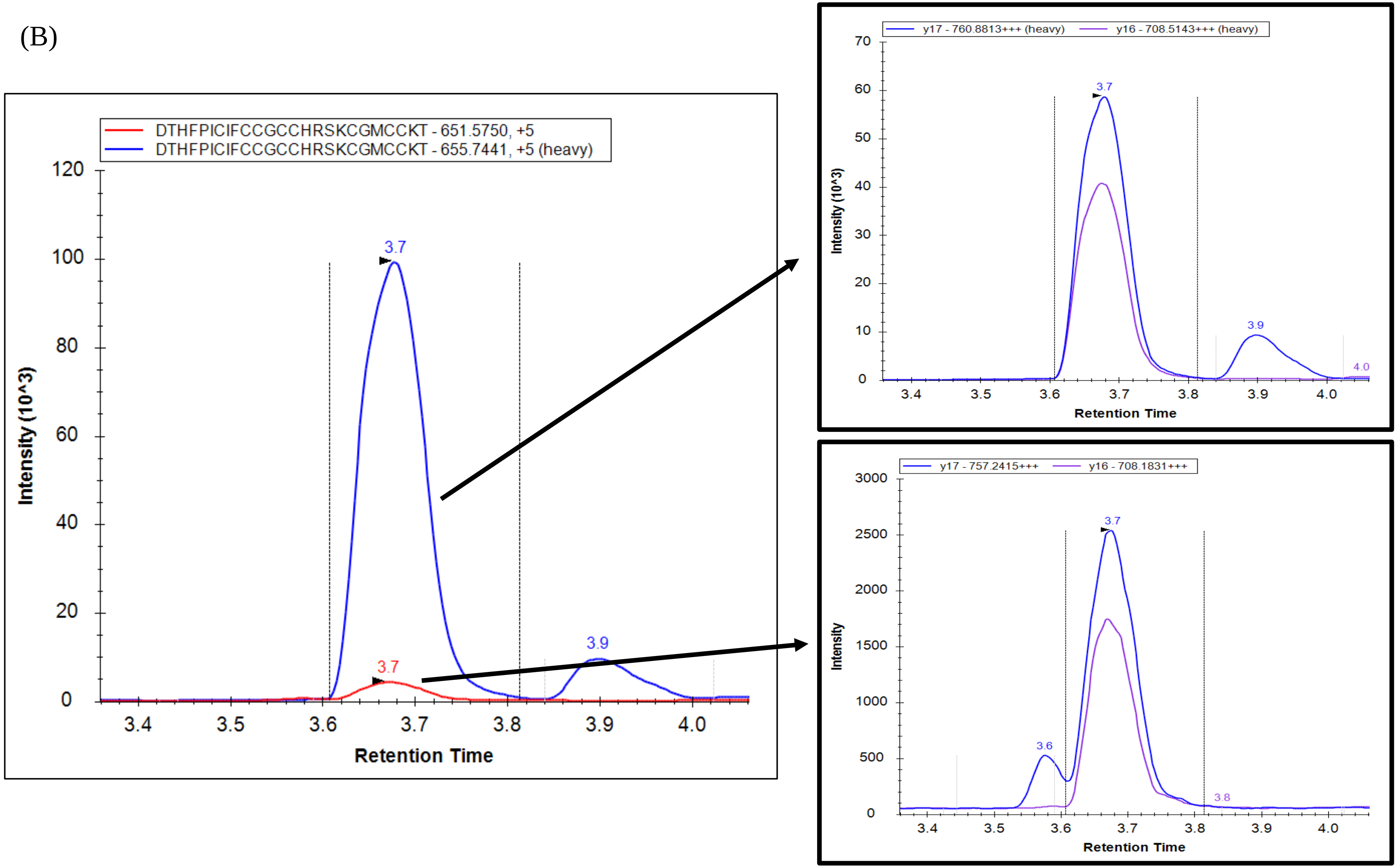

Supplement: Supplementary 2 — Figure 2: the graphs show the extracted ion chromatography (EIC) of hepcidin-I (a) and hepcidin-M (b) obtained from SRM assay using 800 μL of serum sample. Each EIC shows the peak intensity of endogenous- and SI-hepcidin (left) and also shows the peak intensity according to transitions (right). [file 9653747.f2.pdf]
